# Antimicrobial properties of marine fungi from sponges and brown algae of Mauritius

**DOI:** 10.1080/21501203.2021.1895347

**Published:** 2021-04-22

**Authors:** Jessica Mélanie Wong Chin, Daneshwar Puchooa, Theeshan Bahorun, Rajesh Jeewon

**Affiliations:** a Department of Agricultural and Food Science, University of Mauritius, Réduit, Republic of Mauritius; b Department of Biosciences and Ocean Studies, ANDI Centre for Biomedical and Biomaterials Research (CBBR) and University of Mauritius, Réduit, Republic of Mauritius; c Department of Health Sciences, University of Mauritius, Réduit, Republic of Mauritius

**Keywords:** Marine, fungi, mauritius, phylogeny, antimicrobial

## Abstract

**Purpose of the study**: Marine fungi of Mauritius have been poorly studied. There are numerous reports on the bioactive secondary metabolites that are produced by fungi around the world. Yet, research on the molecular characterisation and the pharmaceutical potential of marine fungi in Mauritius is rather scanty.

**Method:** The samples, which consisted of three sponges *Haliclona* sp., *Iotrochota* sp. and *Biemna* sp. and two brown algae *Turbinaria conoides* and *Sargassum portierianum*, were collected in the North of Mauritius during winter. No sporulating structures were observed from the fungal cultures making morphological analysis impossible. The molecular characterisation of the selected isolates was carried out by the amplification of the ITS regions and phylogenetic analysis. The antimicrobial properties were then determined using the disc diffusion and the minimum inhibitory concentration (MIC) assay.

**Results:** Genus level identification was made from molecular data and for some isolates, species-level identification was even possible. Twelve fungi that showed the best antimicrobial properties were identified as *Peniophora* sp., *Aspergillus cristatus, Acremonium* sp., *Cordyceps memorabilis, Aspergillus ochraceus, Biscogniauxia* sp., *Aspergillus keratitidis, Exserohilum rostratum, Chromocleista* sp., *Nigrospora oryzae, Aspergillus flavipes* and *Mycosphaerella*. The lowest MIC result of 0.0098 mg/mL was obtained with *Chromocleista* sp. mycelium extract against *Staphylococcus aureus*. The MIC of the mycelium extracts was lower than the broth extracts for most isolates indicating that the antimicrobial compounds are not secreted.

**Conclusion:** Marine fungi from the Mauritian waters have immense potential in the search for natural products against antibiotic-resistant bacteria.

## Introduction

Antimicrobial resistance can be described as “when bacteria, viruses, fungi and parasites change over time and no longer respond to medicines making infections harder to treat and increasing the risk of disease spread, severe illness and death” (WHO 2020). Antibiotic-resistant bacteria pose a threat to the global health sector as the commonly used antibiotics become less effective. The causes of antimicrobial resistance are the overuse or inappropriate use of antibiotics, lack of hygiene, poor prevention and control of infection, delay in diagnosis and lack of medicines. Therefore, the search for new antimicrobials is indispensable (Christaki et al. 2019). The marine environment has long been screened for antimicrobial compounds. This represents a source of bioactive metabolites with antimicrobial, antiviral, anti-cancer, anti-inflammatory and anti-fouling compounds (Barbosa et al. 2020). Novel antimicrobial compounds have been reported from various marine organisms such as sponges, algae and corals as well as their symbionts (Stincone and Brandelli 2020; Wang et al. 2020). Subramani et al. (2013) isolated a *Penicillium* sp. from the sponge *Melophus* sp. in the Fiji Islands. This isolate produced citrinin which acted against vancomycin-resistant *Enterococcus faecium*. The compounds brevianamide M, 6,8-di-*O*-methylaverufin and 6-*O*-methylaverufin were isolated from the algal endophyte *Aspergillus versicolor*. This endophyte was recovered from the brown algae *Sargassum thunbergii* by Miao et al. (2012) and the three compounds had antimicrobial activities against *Escherichia coli* and *S. aureus*.

Marine sponges are sessile filter feeders that belong to the phylum Porifera. They have a crucial role in nutrient cycling and provide habitat to various organisms. Archaea, bacteria and eukaryotes reside in sponges and make up to 40–60% of their volume (Bart et al. 2020). Sponges are of great interest in the pharmaceutical industry as they have the ability to produce diverse bioactive metabolites that serve as defence mechanism. Unfortunately, a large quantity of the sample is required to extract a small amount of the metabolites. Sponge-associated fungi are also producers of unique metabolites with antimicrobial potentials. Unlike sponges, they can be cultured in the laboratory and large quantities of the metabolite can be extracted (Bovio et al. 2018). Several studies have shown that sponge-associated fungi produce antiviral, antimicrobial, antifungal, antiprotozoal, anti-inflammatory, anticancer and antioxidant compounds (Lekshmi et al. 2020).

Macroalgae are aquatic autotrophic multi-cellular organisms that can be divided into three groups notably red, green and brown algae. Brown algae belong to the class Phaeophyceae and contain the pigment fucoxanthin which gives them a greenish-brown colour. They are used as food in some parts of the world and are habitats for marine organisms (Rushdi et al. 2020). Fungi have been reported as parasites, saprophytes or endophytes in macroalgae. Endophytic fungi spend part of their life cycle inside the tissues or organs of their host without the appearance of any symptoms. A wider range of endophytes have been recovered from red and brown algae as compared to green algae (Suryanarayanan 2012). Endophyte species diversity in macroalgae is affected by environmental factors, season, host species and age (Suryanarayanan and Johnson 2014). Algae endophytes are also important producers of secondary metabolites with great pharmaceutical potentials (Kamat et al. 2020b).

The objectives of this study are:

1. To isolate marine fungi from sponges and brown algae collected around Mauritius
2. To identify the marine fungi based on the Internal Transcribed Spacer (ITS) regions of the ribosomal DNA and phylogenetic analyses
3. To assess the antimicrobial potential of the marine fungi by disc diffusion and MIC assay

## Methodology

### Sample collection

The samples were collected at two locations in the North of Mauritius on 13 August 2018. The sponge *Haliclona* sp. and two brown algae *Turbinaria conoides* and *Sargassum portierianum* were collected at Point-aux-Piments (coordinate: 20°3′46″ S; 57°30′47″ E) while the sponges *Iotrochota* sp. and *Biemna* sp. were collected at Melville (coordinate: 20°1′34″S; 57°42′8″E). The samples were collected at a depth of 1–2 m and placed in Ziploc bags filled with seawater. They were kept on ice during transport and processed within 24 hours. The sponges were identified using the World Porifera Database (Van Soest et al. 2019) while the brown algae were identified using the AlgaeBase (Guiry and Guiry 2019). The physical parameters (pH, temperature and salinity) were recorded during sample collection.

### Isolation of marine fungi

The samples were rinsed with autoclaved seawater to remove debris and sand. Surface sterilisation was carried out to remove epiphytic microorganisms. The samples were cut and dipped in 70% ethanol for 30 s followed by triple rinses in autoclaved seawater. Two-hundred microlitres of the last rinsing water was plated to check for efficiency of sterilisation (Venkatachalam et al. 2015; Bovio et al. 2019). The samples were allowed to dry on sterile paper towels and cut into smaller pieces of 5x5mm^2^. Five pieces of each sample were plated onto Potato Dextrose Agar (PDA), Sabouraud Dextrose Agar (SDA), Malt Extract Agar (MEA), Glucose Yeast Extract Agar (GPY) and Seawater Agar (SWA) supplemented with 1mg/mL chloramphenicol (Kossuga et al. 2012). The media were prepared with filtered autoclaved sea water. The plates were incubated for 4 weeks in the dark at room temperature (25 ± 2°C). They were observed daily and hyphal tips growing from the edges of the sample were sub-cultured. Pure cultures of the morphologically different fungi were made by hyphal tipping. Thirty-seven isolates (13 algal endophytes and 24 sponge-associated fungi) were selected for further studies based on their fast-growing nature or the production of pigments.

### Molecular characterisation of marine fungi

#### DNA extraction, PCR and phylogenetic analysis

The DNA was extracted using the CTAB method by Jeewon et al. (2013). The ITS1-5.8SrRNA-ITS2 regions of the ribosomal DNA were amplified. PCR reactions were carried out in 25 µl reaction volumes and comprised of 2 µl DNA template, 1 µl of 10 µM primers ITS5/ITS4 (White et al. 1990), 12.5 µl of 2X PCR Mastermix (Thermo Scientific^TM^) and 8.5 µl nuclease-free water. The PCR conditions were as follows: initial denaturation at 95°C for 3 min, 35 cycles of denaturation at 95°C for 1 min, annealing at 52°C for 50 s, elongation at 72°C for 1 min and final extension at 72°C for 10 min. The PCR products were checked on 1.5% agarose gels in 0.5X TBE (Tris/Borate/EDTA) at 90 V. They were sent for purification and sequencing at Inqaba Biotechnical Industries (Pty) Ltd, South Africa.

The ITS sequences were submitted to GenBank to obtain accession numbers. The sequences were compared to those available on the National Centre for Biotechnology Information (NCBI) by using the Basic Alignment Search Tool (BLAST). Closely related sequences were retrieved and multiple alignment of the query sequence and related sequences were made using Clustal W in MEGA 7 (Thompson et al. 1994). The phylogenetic trees were constructed in PAUP 4.0 (Swofford 2002) using the Neighbour-Joining method. Distance matrix was generated based on the Kimura’s two parameter model (Kimura 1980). Ambiguously aligned regions, gaps and missing data were removed from the alignment. Evolutionary distances were computed in the number of base substitutions per site. Robustness of the tree was evaluated based on bootstrap values of the 1000 replicates. Branches with less than 50% bootstrap replicates were collapsed.

### Extraction of secondary metabolites

A plug of 10-day old mycelium (6 mmX6mm) was inoculated into 100 mL of seawater PDB. The culture was allowed to grow for 14 days on an orbital shaker at 120rpm to stimulate growth. After 14 days, the broth was separated from the mycelium by vacuum filtration.

The broth was collected and an equal amount of ethyl acetate was added. The extraction was carried out twice with the same volume of solvent. The ethyl acetate was collected after 48 h and evaporated at 40°C. The crude extract was re-suspended in dimethyl sulphoxide (DMSO) to make a stock solution of 20mg/mL (Sharma et al. 2016).

The mycelium was weighed and crushed in ethyl acetate in the ratio of 1:2. It was allowed to macerate for 48 h and the ethyl acetate was evaporated using a rotary evaporator at 40°C. A stock solution of 20 mg/mL was made in DMSO (Handayani et al. 2015).

### Antimicrobial property of fungal extract

#### Disc diffusion assay

The disc diffusion assay was used to assess the antimicrobial properties of the fungal extracts against six human pathogenic bacteria- the three Gram-positive bacteria *Bacillus cereus* (ATCC 10876), *Enterococcus faecalis* (ATCC 29212) and *Staphylococcus aureus* (ATCC 29213) and the three Gram-negative bacteria *Enterobacter cloacae* (ATCC 13047), *Escherichia coli* (ATCC 25922) and *Salmonella typhimurium* (ATCC 14028). The bacteria were allowed to grow overnight in Mueller Hinton Broth (MHB) at 37°C. The cultures were adjusted to the 0.5 McFarland standard with the bacterial suspension being of 1.5 × 10^8^ colony-forming unit (CFU) (absorbance of 0.08–0.1 at 600 nm). One hundred microlitres of bacteria inoculum was spread onto Mueller-Hinton Agar (MHA). Sterile filter paper discs (6 mm) containing 10 µl of the fungal extracts were placed onto the agar plates. The plates were incubated at 37°C for 24 h and the zone of inhibition was measured to the nearest mm (Subramaniam et al. 2020).

#### Broth microdilution assay

The broth microdilution assay was used to determine the minimum inhibitory concentration (MIC) of the extracts. One hundred microlitres of MHB was placed in each well of the 96 well microplate. One hundred microlitres of fungal extract were dispensed in the first well and were two-fold diluted by transferring 100 µl from one well to another until the end of the plate. One hundred microlitres of bacterial inoculum was then added to each well. The plates were incubated at 37°C for 24 h. Twenty microlitres of 0.2 mg/ml of iodonitrotetrazolium violet (INT) was added to each well to determine the MIC. The MIC was taken as the concentration of fungal extract for which no colour change was observed (Öztürk et al. 2019). Results were analysed using a two-way ANOVA in Minitab 17 to determine if there was a significant difference between the extracts.

## Results

### Sample collection and Isolation of marine fungi

**Figure 1.**
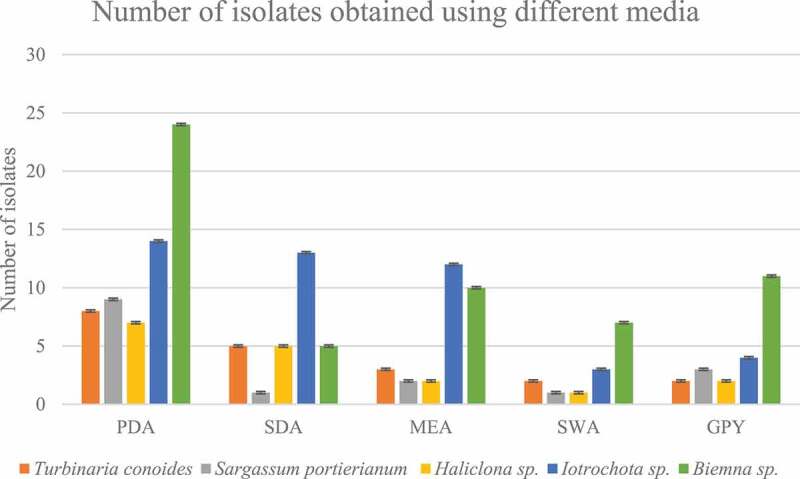
Number of isolates recovered from each sample using different media

The physical parameters on the collection day at Pointe-aux-Piments were temperature 25.4°C, pH 7.87 and salinity of 3.47% whereas at Melville the physical parameters were temperature 24.5°C, pH 7.94 and salinity of 3.50%. A total of 20 isolates were isolated from *T.conoides*, 16 isolates from *S.portierianum*, 17 isolates from *Haliclona* sp., 46 isolates from *Iotrochota* sp. and 57 isolates from *Biemna* sp. It was on PDA that most isolates were recovered (39.74%). The same percentage of isolates were recovered from SDA and MEA (18.59%), 14.10% of isolates were recovered from GPY while the lowest amount were recovered from SWA (8.97%) as shown in Figure 1.

### DNA-based identification and phylogenetic analysis

Most of the isolates were identified up to the genus level using the ITS regions of the ribosomal DNA. The percentage identity of the closest match ranged from 85.60% to 100.00%. The closest BLAST result match, the percentage identity, the percentage query cover as well as the accession numbers of the selected fungi are shown in Table 1.

**Table 1.** BLAST result search of the selected marine fungi

Isolate	Host	Genus	Accession Number	Description of closest match from BLAST search	Percentage Identity	Percentage Query Cover
F1	*S. portierianum*	*Peniophora*	MW187733	*Peniophora* sp. AR-2010 isolate ATT289 (HQ607923.1)	96.10%	97%
F2	*T. conoides*	*Aspergillus*	MW187734	*Aspergillus cristatus* strain DUCC5705 (MT582745.1)	100.00%	97%
F3	*S. portierianum*	*Curvularia*	MW187735	*Curvularia verruculosa* isolate TZ1(MF945600.1)	100.00%	97%
F4	*Biemna* sp.	*Montagnula*	MW187736	*Montagnula opulenta* strain 5-F13 (MW081387.1)	95.52%	99%
F6	*Iotrochota* sp.	*Xylaria*	MW187737	*Xylaria* sp. Id-BL131 (LC505186.1)	100.00%	98%
F7	*Haliclona* sp.	*Pyrenochaeta*	MW187738	*Pyrenochaeta* sp. 14009 (EU750693.1)	99.24%	97%
F8	*Haliclona* sp.	*Cladosporium*	MW187739	*Cladosporium delicatulum* (MN644691.1)	100.00%	96%
F9	*Biemna* sp.	*Paraconiothyrium*	MW187740	*Paraconiothyrium* sp. strain PNB15 1B1 (MH268018.1)	98.63%	54%
F10	*S. portierianum*	*Pseudopithomyces*	MT799844	*Pseudopithomyces maydicus* strain MFLUCC 17–0028 (MG545071.1)	98.60%	96%
F12	*T. conoides*	*Acremonium*	MT799845	*Acremonium* sp. 3 MMW-2015 strain OUCMBI110137 (KP269047.1)	98.18%	85%
F13	*S. portierianum*	*Curvularia*	MT799850	*Curvularia lunata* isolate AX-MGMH-2 (MG783400.1)	99.12%	98%
F14	*Iotrochota* sp.	*Trichoderma*	MW187741	*Trichoderma breve* isolate TWS48Abf(b) (MN400089.1)	99.67%	99%
F15	*S .portierianum*	*Roussoella*	MW187742	*Roussoella* sp. 1 NV-2015 (LT796863.1)	99.45%	98%
F16	*T. conoides*	*Cordyceps*	MT799846	*Cordyceps memorabilis* strain CCRC 32218 (AY245632.1)	99.47%	94%
F17	*Biemna* sp.	*Pleosporales*	MW187743	*Pleosporales* sp. ICMP 18095 (HM116753.1)	99.82%	97%
F19	*S. portierianum*	Clonostachys	MW187744	*Clonostachys rosea* 197WS (MG396999.1)	99.64%	97%
F20	*Iotrochota* sp.	*Fusarium*	MW187745	*Fusarium keratoplasticum* strain WM 06.858(KP132216.1)	100.00%	96%
F21	*Biemna* sp.	*Phomopsis*	MW187746	*Phomopsis* sp. DZ27 (EU236704.1)	99.12%	98%
F22	*Haliclona* sp.	*Fusarium*	MW187747	*Fusarium chlamydosporum* strain DEB15 (KF918598.1)	99.27%	98%
F23	*Biemna* sp.	*Paraconiothyrium*	MW187748	*Paraconiothyrium* sp. strain PNB15 1B1 (MH268018.1)	98.64%	55%
F24	*Iotrochota* sp.	*Eutypella*	MW187749	*Eutypella* sp. strain Eef-15 (MK120868.1)	95.38%	97%
F25	*Iotrochota* sp.	*Aspergillus*	MW187750	*Aspergillus ochraceus* isolate TN-26 (KX610750.1)	93.23%	96%
F26	*Iotrochota* sp.	*Auxarthron*	MW187751	*Auxarthron pseudoreticulatus* (AJ271420.1)	99.50%	96%
F27	*Biemna* sp.	*Parengyodontium*	MW187752	*Parengyodontium album* strain MEFCO54 (MK732105.1)	99.83%	98%
F28	*Iotrochota* sp.	*Biscogniauxia*	MW187753	*Biscogniauxia* sp. B1B0853P152CC977 (KP306930.1)	99.85%	96%
F29	*Iotrochota* sp.	*Aspergillus*	MW187754	*Aspergillus keratitidis* strain KAS7927 (KY980626.1)	99.84%	97%
F30	*Iotrochota* sp.	*Exserohilum*	MW187755	*Exserohilum rostratum* isolate UASBW13 (MN599588.1)	99.66%	96%
F31	*Iotrochota* sp.	*Letendraea*	MW187756	*Letendraea helminthicola* isolate A1S5-12 (KJ774052.1)	99.69%	97%
F33	*Haliclona* sp.	*Exserohilum*	MW187757	*Exserohilum rostrate* (LT984841.1)	100.00%	96%
F34	*Biemna* sp.	*Chromocleista*	MW187758	*Chromocleista* sp. (MN644566.1)	99.81%	97%
F35	*Biemna* sp.	*Diaporthe*	MW187759	*Diaporthe* sp. strain LFIT03 (MK299422.1)	99.28%	96%
F36	*S.portierianum*	*Nigrospora*	MW187760	*Nigrospora oryzae* strain LMMS-15 (KT824761.1)	100.00%	96%
F37	*S. porteirianum*	*Aspergillus*	MW187761	*Aspergillus flavipes* strain E14 (GU566238.1)	99.65%	97%
F38	*S. portierianum*	*Mycosphaerella*	MW187762	*Mycosphaerella* sp. ZJ12-2A (FJ037771.1)	99.47%	98%
F39	*Iotrochota* sp.	*Amesia*	MW187763	*Amesia nigricolor* En10 (MN180855.1)	100.00%	98%
F40	*T. conoides*	*Passalora*	MW187764	Cf. *Passalora* sp. CPC 11876 (GU214642.1)	99.27%	97%
F41	*Iotrochota* sp.	*Phaeosphaeria*	MW187765	*Phaeosphaeriaceae* sp. isolate MBD 3628 (MK595671.1)	85.60%	86%

BLAST results and phylogenetic analysis of the sequences were used to infer fungal identity. Endophytes from the brown algae *T. conoides* were from the Trichomaceae, Hypocreaceae, Cordypitaceae and Mycosphaerellaceae family whereas those from the brown algae *S. portierianum* belonged to the Peniophoraceae, Pleosporaceae, Didymosphaeriaceae, Bionectriaceae, Apiosporaceae, Trichomaceae and Mycosphaerellaceae family. Regarding the sponges, the fungi which were isolated from *Haliclona* sp. belonged to the “Incertae sedis”, Davidiellaceae, Nectriaceae and the Pleosporaceae family. The *Iotrochota* sp. associated fungi were from the Xylariaceae, Hypocreaceae, Nectriaceae, Diatrypaceae, Trichomaceae, Onygenaceae, Pleosporaceae, “Incertae sedis”, Chaetomiaceae and Phaeosphaeriaceae family. The fungi which were isolated from the sponge *Biemna* sp. were from the Montagnulaceae, Didymosphaeriaceae, Pleosporales, Valsaceae, Cordypithaceae, Trichocomaceae and Diaportheceae family.

Isolate F1 was found to be closely related to *Peniophora* sp. (HQ607923.1) with high bootstrap value of 93% (Figure 2(a)). Isolate F2 was found to be related to many *Aspergillus* species with bootstrap value of 87%. F25 was closely related to many *Aspergillus ochraceus* strains with 100% bootstrap value. Isolate F29 was closely related to *Aspergillus keratitidis* with bootstrap value of 87% while isolate F34 was closely related to *Chromocleista* sp. (MN644566.1) with 100% bootstrap value. Isolate F37 was closely related to many *Aspergillus flavipes* with 70% bootstrap value as seen in Figure 2(b). Isolate F3 was closely related to *Curvularia verruculosa* (MH368139.1) strain with 61% bootstrap value while F13 was closely related to *Curvularia lunata* isolate (MG783400.1) with bootstrap value of 76%. Isolates F30 and F33 were related to each other with bootstrap value of 65%. They were closely related to *Exserohilum rostratum* (MN599588.1) too with bootstrap value of 82% (Figure 2(c)). Isolate F4 was closely related to *Montagnula opulenta* (MW081387.1) with bootstrap value of 53% as seen in Figure 2(d). From Figure 2(e), it can be noticed that F28 was related to many *Biscogniauxia* sp. with high bootstrap value of 100%. F6 was also related to two *Xylaria* sp. with bootstrap value of 95%.

**Figure 2.**
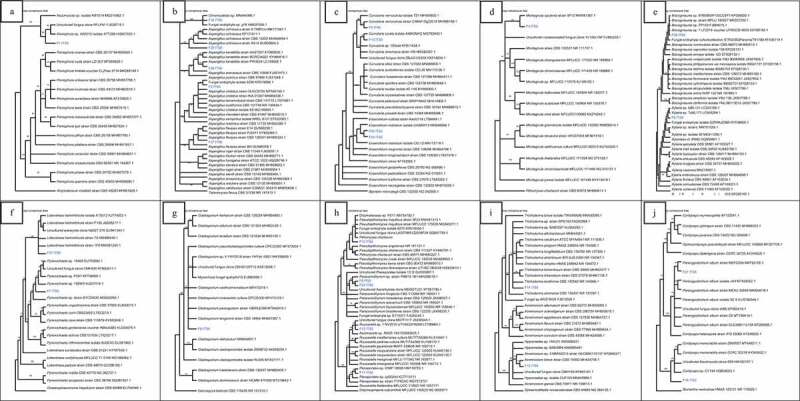
Neighbour-joining trees showing the relationships of isolates with members of their family based on ITS rDNA sequence. (a) Isolate F1 with members of Peniophoraceae. (b) Isolates F2, F25, F29, F34, F37 with members of Trichomaceae. (c) Isolates F3, F13, F30, F33 with members of Pleosporacea. (d) Isolate F4 with members of Montagnulaceae. (e)Isolates F6, F28 with members of Xylariaceae. (f) Isolates F7, F31 with members of “Incertae sedis”. (g) Isolate F8 with members of Davidiellaceae. (h) Isolates F9, F10, F15, F17, F23 with members of Didymosphaeriaceae. (i) Isolates F12, F14 with members of Hypocreaceae. (j) Isolates F16, F27 with members of Cordypitheaceae. (k) Isolates F19 with members of Bionectriaceae. (l) Isolates F20, F22 with members Nectriaceae. (m) Isolates F21, F35 with members Diapothaceae. (n) Isolate F24 with members Diatrypaceae. (o) Isolate F26 with members Onygenaceae. (p)Isolate F39 with members of Chaetomiceae. (q) Isolates F38, F40 with members of Mycosphaerellaceae. (r) Isolate F36 with members Apiosporaceae

Isolate F31 was related to many *Letendraea helminthicola* strains with high bootstrap value of 100% while F7 was related to *Pyrenochaeta* sp. with bootstrap value of 82% (Figure 2(f)). Isolate F8 was related to many species of *Cladosporium* as seen in Figure 2(g). Isolate F9 was found to be closely related to isolate F23. These were related to *Paraconiothyrium* sp. (MH268018.1) with bootstrap value of 67%. Isolate F10 was found to be closely related to two *Pseudopithomyces maydicus* strain. Isolate F15 was related to *Roussoella* sp. (LT796863.1) with bootstrap value of 50% whereas isolate F17 was found to be related to *Pleosporales* sp. (HM116753.1) with bootstrap value of 95% (Figure 2(h)). In Figure 2(i), isolate F12 was closely related to *Acremonium breve* (MH424706.1) with bootstrap value of 71% while F14 was found to be related to many species of *Trichoderma*. F16 was related to two *Cordycceps memorabilis* strain while isolate F27 was closely related to *Parengyodontium album* (MK73205.1) with bootstrap value of 60% (Figure 2(j)).

In Figure 2(k), F19 was closely related to *Clonostachys* sp. (MT215573.1) with bootstrap value of 75%. Isolate F20 was related to two *Fusarium* species with bootstrap value of 65% while F22 was related to many species of *Fusarium* with bootstrap value of 100% (Figure 2(l)). Isolate F21 was closely related to *Phomopsis* sp. (EU236704.1) with bootstrap value of 78% whereas F35 was related to three species of *Diaporthe* with bootstrap value of 100% (Figure 2(m)). In Figure 2(n), isolate F24 was related to three *Eutypella* sp. and two species of *Eutypella* with 100%bootstrap value. Isolate F26 was found to be related to two *Auxarthron pseudoreticulatus* (AJ271420.1, NR111111.1) with bootstrap value of 100% (Figure 2(o)).

Isolate F39 was related to *Amesia nigricolor* (MN180855.1) with bootstrap value of 52% as seen in Figure 2(p). In Figure 2(q), it can be observed that F40 was closely related to *Passalora* sp. (GU214642.1, KX065270.1) with high bootstrap value of 100%. Isolate F38 was also related to three *Mycosphaerella* sp. In Figure 2(r), isolate F36 was related to *Nigrospora oryzae* and *Nigrospora pyriformis* with bootstrap value of 55%.

**Figure 2.**
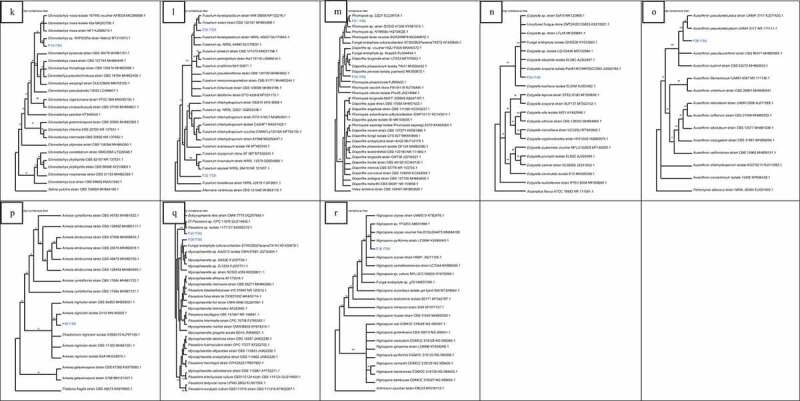
(Continued)

### Antimicrobial activity of marine fungi

**Figure 3.**
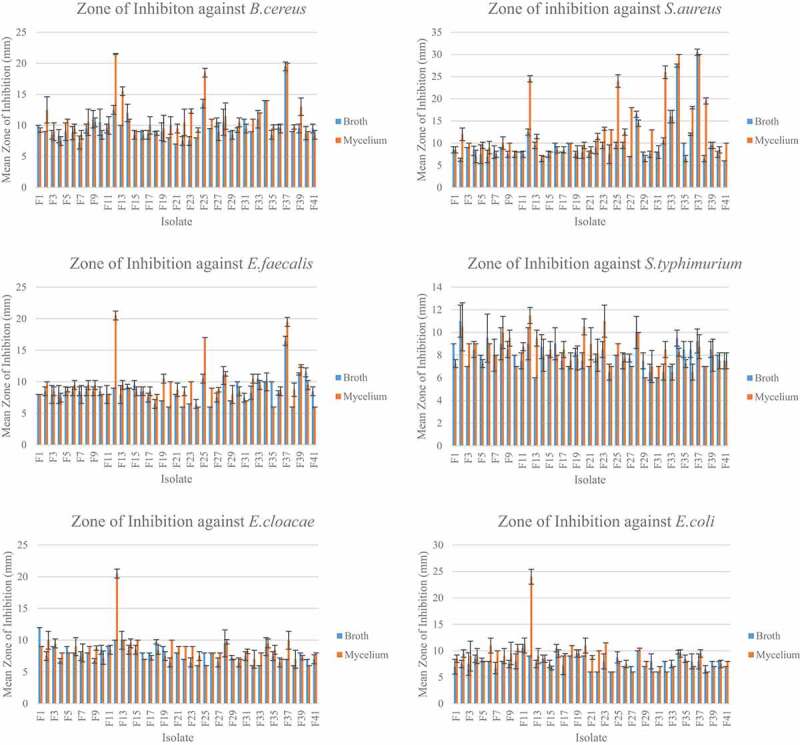
Mean zone of inhibition of broth and mycelium extracts against the six bacteria

The thirty-seven isolates (Figure 3, Supplementary material) were able to inhibit the six bacteria used in this study. There were significant differences between the MIC of the mycelium and the broth extracts as p < 0.05. The MIC assays were performed thrice and the MIC of the extracts was consistent. The mycelium extracts showed better antimicrobial activity as compared to the broth extracts. Overall, the extracts showed better inhibition against Gram-positive bacteria as compared to the Gram-negative bacteria.

The mean zone of inhibition for the broth extracts ranged from 6.00 ± 0.00 mm to 30.5 ± 0.70 mm (Figure 3). Extract F34 had the highest antibacterial activity against *B. cereus* with mean zone of inhibition of 14 ± 0.00 mm. Extract F37 had a zone of inhibition of 30.5 ± 0.71 mm against *S. aureus* while F40 has the largest zone of inhibition of 11.50 ± 0.71 mm against *E. faecalis*. Regarding the Gram negative bacteria, F2 had zone of inhibition of 11.00 ± 1.41 mm against *S. typhimurium*, F1 had zone of inhibition of 12.00 ± 0.00 mm against *E. cloacae* and F16 had zone of inhibition of 10.5 ± 0.71 against *E. coli*. For the mycelium extracts, the mean zone of inhibition ranged from 30.00 ± 0.00 mm to 6.00 ± 0.00 mm. Extract F12 had the highest antibacterial activity with mean zone of inhibition of 21.50 ± 0.71 mm against *B. cereus*. Regarding *S. aureus* and *E. faecalis*, the highest antibacterial activity was with extract F34 and F12 with mean zone of inhibition of 30.00 ± 0.00 mm and 20.50 ± 0.71 mm respectively. Extract F12 had the highest antibacterial activity against the three Gram negative bacteria *S. typhimurium, E. cloacae* and *E. coli*. It had mean zone of inhibition of 11.50 ± 0.71 mm, 20.50 ± 0.71 mm and 24.00 ± 1.41 mm against *S. typhimurium, E. cloacae* and *E. coli* respectively.

**Figure 4.**
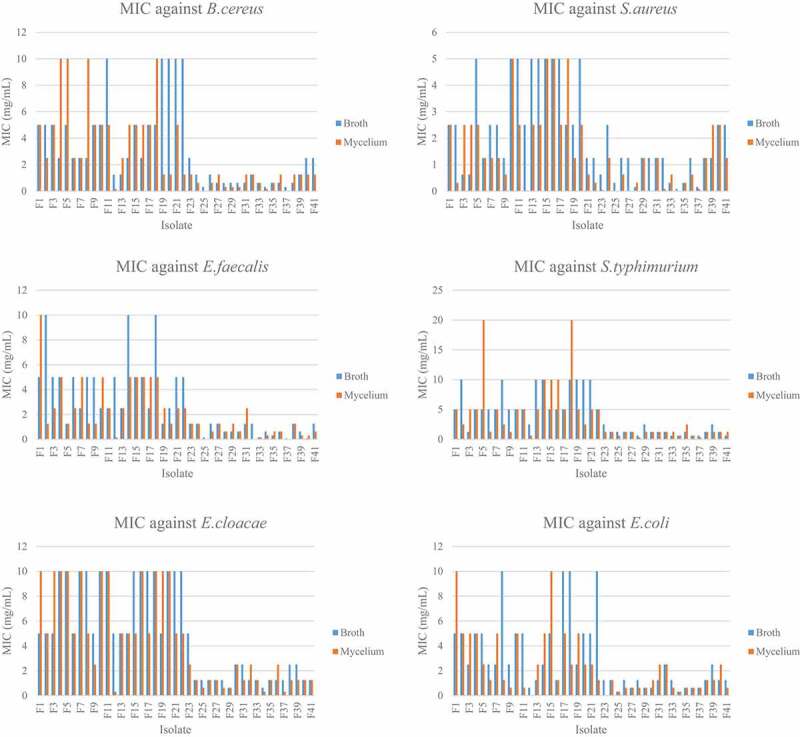
MIC of broth and mycelium extracts against six bacteria

The MIC ranged from 0.078 mg/ml to 10 mg/ml for the broth extracts while it ranged from 0.098mg/ml to 10 mg/ml for the mycelium extracts. For the broth extracts, F25, F34 and F37 had the lowest MIC of 0.31 mg/ml against *B. cereus*. The lowest MIC was 0.078 mg/ml for extract F34 and F37, F40 against *S. aureus* and *E. faecalis* respectively. For the Gram negative bacteria, extracts F33, F34, F36, F37 and F41 had the lowest MIC of 0.625 mg/ml against *S. typhimurium*. F29 and F34 extracts have the lowest MIC of 0.625 mg/ml against *E. cloacae*. For *E. coli*, the extracts F25 and F34 had the lowest MIC of 0.31 mg/ml (Figure 4). Regarding the mycelium extracts the MIC ranged from 0.0098 mg/ml to 10 mg/ml for the 37 extracts tested. The extracts F25 and F37 had the lowest MIC of 0.020 mg/ml against *B. cereus*. Extract F34 had the lowest MIC of 0.0098 mg/ml against *S. aureus* while extracts F25 and F37 had the lowest MIC of 0.020 mg/ml against *E. faecalis*. The lowest MIC against *S. typhimurium* was of 0.31 mg/ml and was obtained with extracts F28 and F38. The MIC of 0.31 mg/ml was also obtained with extracts F12, F34 and F37 against *E. cloacae*. With the bacteria *E. coli*, the lowest MIC was of 0.039 mg/ml and was with extract F23.

## Discussion

### Isolation of marine fungi

The number of algal endophytes was in accordance with the study by Venkatachalam et al. (2015). However, it was lower than the number of fungi recovered by Zuccaro et al. (2008). The brown algae *Fucus serratus* contained 336 isolates representing 35 genera of the Ascomycota and Zygomycota. Regarding the sponge-associated fungi, fewer were isolated from *Haliclona* sp. as compared to those from *Iotrochota* sp. and *Biemna* sp. The number of isolates recovered depends on many factors such as the environmental conditions and the type of host. These factors can account for the difference in the number of fungi isolated from the three different sponges. Regarding the agars used in this study, nutritionally rich ones yield a greater number of isolates as compared to the nutritionally depleted ones. The SWA yielded the smallest number of isolates as it does not contain many nutrients. On the contrary, more isolates were able to grow on the PDA, MEA and SDA as they are rich in nutrients. The good choice of media and growth conditions is primordial in isolation of marine fungi (Kossuga et al. 2012).

### Molecular characterisation & antimicrobial properties

All of the selected fungal extracts were able to inhibit microbial growth. The results indicate that the antimicrobial metabolites of the selected fungi were both extracellular and intracellular. However, the mycelium extracts showed better antimicrobial properties overall. A study by Synytsya et al. (2017) demonstrated that fungal mycelium extracts have good antimicrobial activity. Secondary metabolite production depends on many factors when the fungi are grown in the lab. These include the fungal strain, the growth medium and conditions (VanderMolen et al. 2013; Synytsya et al. 2017; Overy 2017; Petersen et al. 2019). Potato Dextrose Broth is commonly used as medium for secondary metabolite production as it contains high glucose concentration, a condition favourable for antimicrobial metabolite production (Miao et al. 2006; Aiatwani 2016). Seawater was also added to the culture as this incites the production of unique compounds (Wang et al. 2011). The seawater applies a selective pressure that upregulates the production of certain metabolites leading to the discovery of unique natural products (Wang et al. 2011; Overy 2017). The solvent which was used in this study was ethyl acetate. This is often used in fungal secondary metabolite extraction as it is able to extract various compounds with bioactive potentials. This was stated by Kamat et al. (2020a) who examined the chemical composition of the ethyl acetate extract of a marine algae endophyte by GC-MS. The fungal secondary metabolites were more active against Gram-positive bacteria as compared to Gram-negative bacteria. This is due to the difference in their cell wall structure.

Isolate F1 was identified as *Peniophora* sp. Members of this genus have been recovered as endophytes from marine habitats and are therefore adapted to thrive in these conditions. *Peniophora* sp. was recovered from rhizosphere soil, Brazilian sponge *A.viridis* and the mangrove *Bruguiera gymnorrhiza* in South China (Gonçalves et al. 2015; Brenelli et al. 2019; Li et al. 2020). This species has not been reported from macroalgae in Mauritius. The *Peniophora* sp. isolated in this study showed good antimicrobial properties, especially against *E. cloacae*. A study by Manju (2019) showed that species of *Peniophora* produce bioactive metabolites with reported antioxidant, anti-tumour and antibacterial properties. The *Aspergillus* genus is ubiquitous and many have been isolated as endophytes, saprophytes and even pathogens in the marine environment. This genus is a well-known producer of secondary metabolites with immense bioactive potentials (Blunt et al. 2016; Nicoletti and Vinale 2018; Khalil et al. 2020). Marine-derived fungi that are repeatedly recovered belong to the genera *Aspergillus*. In this study, it was the most abundant genera from brown algae and sponges. Isolate F2 was related to three *Aspergillus* species and it was identified as *Aspergillus cristatus*. Isolate F25 was identified as *Aspergillus ochraceus* while isolate F29 was identified as *Aspergillus keratitidis*. The different species of *Aspergillus* recovered from the Mauritian waters have shown good antimicrobial properties against the bacteria used in this study. Over 120 natural bioactive compounds are produced by *Aspergillus* species (Lee et al. 2013). *A. ochraceus* has shown good antimicrobial properties in a study by Attia et al. (2019). LC-MS studies have shown that it produces the previously reported antimicrobial compounds versicolin, terreic acid, fumigatin, aspyrone, 4-hydroxymellin and terremutin. Isolate F37 was also identified based on its 0.35% nucleotide difference with other *Aspergillus flavipes* strains. Guo et al. (2019) reported the antimicrobial properties of a marine *Aspergillus flavipes* against the aquatic pathogen *Vibrio harveyi*. Its bactericidal activity was due to questin which destroys the bacterial cell wall and membrane causing leakage of cellular components.

The genus *Acremonium* contains species that are saprophytes, pathogens as well as endophytes (Summerbell et al. 2018; Han et al. 2020). Isolate F12 was identified as a member of this genus as it had 98.18% sequence similarity with *Acremonium* sp 3 MMW-2015 strain (KP269047.1) which was recovered as endophyte from the green macroalgae *Codium fragile* from the coast of Qingdao, China (Wang et al. 2018). The genus *Acremonium* has also been reported from sponges, mangroves and seawater (Chen et al. 2012; Julianti et al. 2012; Chung et al. 2019). *Acremonium* species are producers of bioactive compounds (Hsiao et al. 2020; Rahim 2020) and the strain isolated in this study had very promising antimicrobial properties. The marine *Acremonium persicinum* isolated by Luo et al. (2019) produced sideromycins, which are known for the low MIC values in antimicrobial tests. The *Cordyceps* genus is known as being entomopathogenic and many species are parasites to insects (Shrestha et al. 2016). F16 was identified as *Cordyceps memorabilis*. Reports on terrestrial endophytic *Cordyceps* are numerous but those on marine-derived ones are lacking. This study demonstrates that *Cordyceps memorabilis* exists in the marine environment as an algal endophyte. Members of this genus have been reported as producers of bioactive secondary metabolites (Chen et al. 2020) and the strain isolated in this study forms part of this group. The five new antimicrobial anthraquinones, morakotins A-E, have been isolated from *Cordyceps morakotii* by Wang et al. (2018). These compounds showed strong antimicrobial properties with MIC 3.13–25 µg/ml against *B. cereus* and *S. aureus.*

Isolate F28 formed a clade with many *Biscogniauxia* sp. with high bootstrap support of 100%. The isolate *Biscogniauxia* sp. B1b0856P152CC396 (KP306931.1), which is closely related to isolate F28, was recovered from a sponge from the Caribbean and the Pacific of Panama (Bolaños et al. 2015). This indicates that this genus can be sponge-associated although it is known for being endophytes and pathogens in plants (Costa et al. 2020). Additionally, *Biscogniauxia* sp. has been recovered from sediments and hard substrates/rock substrates in the marine environment (Wu et al. 2016; Fouillaud et al. 2017). A study by An et al. (2020) has shown that *Biscogniauxia* sp. endophyte has antimicrobial activity against two pathogenic bacteria. Another study by Liu et al. (2019) explained that *Biscogniauxia* sp. was able to produce phthalide derivatives which accounted for its anti-acetyl and antimicrobial activities. This explains why the isolate F28 in this study showed good antimicrobial properties. Isolate F33 was identified as *Exserohilum rostratum* as it was closely related to *Exserohilum rostratum* strain UASBW13. The genus Exserohilum was recovered as endophyte, saprophyte and parasite in plants (Bagur et al. 2020). The isolation of this species from the sponge *Haliclona* sp. in this study indicates that it can be adapted to live in the marine environment too. A marine *Exserohilum rostratum* was isolated in cyanobacterial mat by Tang et al. (2004) proving that this species is adapted to survive in marine conditions. The latter also produced rostratins A-D with bioactive properties hence explaining the antimicrobial properties of the isolate F33 in this study.

Isolate F34 was identified as *Chromocleista* sp. as it was closely related to the *Chromocleista* sp. (MN644566.1). The *Chromocleista* sp. (MN644566.1) was recovered from *Sporobolus pumilus* in the Louisiana coastal marshes. Another *Chromocleista* sp. was isolated by Park et al. (2006) from a deep-water sediment sample collected in the Gulf of Mexico. This isolate produced the three new compounds, p-hydroxyphenopyrrozin and deketopiperazines, as well as four known compounds. The isolate F34 extract in this study was among the best antimicrobial agents indicating that it can also produce bioactive metabolite. Isolate F36 was identified as *Nigrospora oryzae* as it had 100% sequence similarity with *Nigrospora oryzae* strains. *Nigrospora*is a genus that contains phytopathogens, endophytes and saprophytes on different hosts (Hao et al. 2020). Marine *Nigrospora oryzae* have been isolated from sponges, soft coral, sea fan and seaweeds (Trisuwan et al. 2008; Sun et al. 2014; de Felício et al. 2015; Ding et al. 2016a). Therefore, this fungus exists in the marine environment. Secondary metabolites have been isolated from marine *Nigrospora oryzae* with antimicrobial, antioxidant and anti-tumour properties (Ding et al. 2016b). A marine-derived *Nigrospora oryzae* produced nigrospine (alkaloids) and citrinins in a study by Dong et al. (2014). These bioactive compounds are known to possess antimicrobial properties. This explains why the isolate F36 possessed good antimicrobial properties against the bacteria in this study. Isolate F38 was identified as *Mycosphaerella* sp. as it was closely related to many *Mycospaherella* species. This genus can be found in plants as endophytes, saprophytes and parasites (De Queiroz and Santana 2020). Studies have isolated *Mycosphaerella*sp. from brown algae *Ascophyllum nodosum*, sediment and mangroves (Fries 1979; Lee et al. 2019; Qiu et al. 2019). It exists in the marine environment where it produces secondary metabolites with biological properties (Qiu et al. 2019). The *Mycosphaerella* sp. recovered by Lee et al. (2019) produced the antibacterial usnic acid congeners. These possessed strong antibacterial activity against the six bacteria used in the study.

## Conclusion

The Mauritian waters contain microorganisms that are awaiting to be discovered and studied. The algae and sponges that live in the coastal waters contain a plethora of fungi that produce interesting secondary metabolites. The marine fungi isolated in Mauritius showed promising antimicrobial properties and the compounds that are responsible for this need to be identified. These can be useful in the search for antimicrobials against resistant bacteria. Reports on the antimicrobial potentials of mycelium and broth extracts of marine fungi in Mauritius are scarce. The latter have not been properly characterised and need to be identified using DNA-based methods. Proper identification is a must in natural product research. The ITS rDNA regions are useful to ascertain genus identity but have to be used carefully in species-level identification.
